# The effect of regular running on body weight and fat tissue of individuals aged 18 to 65

**DOI:** 10.1186/s40101-023-00348-x

**Published:** 2023-11-30

**Authors:** Petr Kutac, Václav Bunc, Marek Buzga, Miroslav Krajcigr, Martin Sigmund

**Affiliations:** 1 Department of Human Movement Studies, University of Ostrava, Ostrava, 701 03 Czech Republic; 2 Faculty of Education, Charles University, Praha 6, Praha, 162 52 Czech Republic; 3 Application Centre BALUO, Faculty of Physical Culture, Palacky University, Olomouc, 779 00 Czech Republic

**Keywords:** Running, Inactive individuals, Weight status, Body fat, Visceral fat

## Abstract

**Background:**

Age and reduction in performed physical activity cause physiological changes that include an increase in body fat (BF) and visceral fat (VF) during aging. These parameters, together with increased body mass (BM), are some of the risk factors of several noninfectious diseases. However, changes in body composition can be influenced by regular physical activity. Running is a suitable, accessible, and the most effective physical activity cultivating people. The objective of this study is to investigate the effects of long-term, regular PA, specifically recreational running, on changes in body composition among recreational adult runners covering a weekly distance of at least 10 km, compared with inactive adult individuals within the same age bracket.

**Methods:**

The study included 1296 runners and inactive individuals (691 male and 605 female), divided into 5 age groups: 18–25, 26–35, 36–45, 46–55, and 56–65 years. Runners are as follows: ran ≥ 10 km/week, and inactive is as follows: did not follow the WHO 2020 physical activity recommendations. The measured parameters included BM, BF, and VF. To check statistical significance, the Mann–Whitney *U*-test was used. Practical significance was assessed using the effect of size.

**Results:**

All age groups of runners were selected to include individuals who run at least 10 km per week. In fact, they ran, on average, from 21.6 to 31.4 km per week in relation to age and showed significantly lower values of BM, BMI, BF, and VF (*p* < 0.05) than inactive individuals. Exceptions included insignificant differences (*p* > 0.05) in BM and BMI in males in the age category of 18–25 and in females in the age category of 18–25 and 26–35.

**Conclusion:**

The selected runners had to run at least 10 km per week. Their actual average volume was significantly higher (from 21.6 to 31.4 km/week), and the results showed that it could lead to significantly better body composition values. It may lead to significant changes in body mass, body fat, and visceral fat. It may meet the contemporary societal expectations for physical activities that are both achievable and effective at the lowest possible volume.

**Supplementary Information:**

The online version contains supplementary material available at 10.1186/s40101-023-00348-x.

## Introduction

Movement is a fundamental biological need, and the ability to realize it is one of the basic prerequisites for healthy aging. Long-term active individuals are able to maintain their independence even in old age. The question that arises pertains to how regular physical activity (PA) impacts various body composition parameters. Functional and physiological changes occur during aging in dependence on the individual’s behavior [1]. Such changes also include changes in body composition, characterized by increased adiposity and loss of fat-free mass (FFM), and thus, muscle mass, which, among other things, is the result of insufficient PA volume [2, 3]. Such changes can lead to the occurrence of sarcopenia, characterized by low levels of three parameters: muscle strength, muscle quantity/quality, and physical performance [4]. Sarcopenia occurs more frequently in individuals 60 years and older [5]. Sarcopenia limits everyday function and contributes to invalidity, decreasing one’s independence and self-reliance [6, 7]. However, the results of a study by Bautmans et al. [8] showed that a regular loss of FFM in inactive participants, ranging from 0.34 to 1.28%, starts at the age of 30 years. Changes occur in both men and women [9–12]. Changes in women are uneven due to menopause [13]. The results based on data from the Study of Women’s Health Across the Nation (SWAN) [13] showed a considerable increase in body fat (BF) in women, accompanied by a decrease in fat-free mass (FFM) about 2 years prior to the final menstrual period. After that, the FFM decreases, and the BF increases evenly.

Assessing body composition is a critical aspect of an individual’s health diagnosis. Merely evaluating BM and BMI falls short in providing insights into the prerequisites for PA. This requires an assessment of individual BM fractions and their distribution, primarily focusing on adipose tissue and muscle mass. Obesity is characterized by an increased amount of body fat (BF), which is recognized as a risk factor for a range of diseases (diabetes, cardiovascular diseases, musculoskeletal disorders) [14]. Currently, increased attention is being paid to visceral fat (VF). Elevated levels of VF are considered a more substantial risk factor for severe metabolic and cardiovascular diseases compared to BF or BMI [15–17]. VF is more metabolically active and secretes cytokines and hormones that exert metabolic disturbances such as insulin resistance and chronic low-grade inflammation at a higher rate [18].

Regular PA is a variable and manageable parameter, making it a fundamental requirement for maintaining ideal body composition and optimal values for body fat and muscle mass parameters. An adequate volume and quality of muscle mass empower individuals to engage in physical activities even at an older age. Therefore, regular PA is considered a preventive factor of many chronic, noninfectious diseases such as cardiovascular diseases, cancer, type 2 diabetes, bone health degradation, and increased disability [19–21]. Many of these diseases are major factors in the mortality rate [22, 23]. A regular PA creates conditions for a better health condition which may lead to lower mortality. The results of a study by Reimers and colleagues [24] indicate that mortality decreases by 30 to 35% in physically active individuals compared to inactive individuals. Regular PA not only improves quality of life but also extends life expectancy without chronic diseases. The results of the health analysis of adults aged 50 to 75 years with a variety of PA levels showed that strongly active male and female individuals lived, on average, 6.3 years longer in good health condition and 2.9 years longer without chronic diseases than inactive individuals [25]. According to the study by Alvero-Cruz et al. [26], physically active individuals—master athletes (athletes at the age of 40 and above who regularly participate in competitions), are considered a model of healthy aging as they can reduce the age-related decline of physiological abilities compared to sedentary individuals [26]. Regular PA is also important in the prevention of obesity. This is confirmed by including regular PA in the pillar management of people with obesity [27]. It is suitable to include a PA that requires the participation of large muscle groups, such as running, in people who are not obese (according to the WHO: BMI < 30.0 kg/m^2^). Compared with other PAs, running is easily accessible, and its implementation does not require any special equipment or special conditions. Running is thus a very popular type of PA. The participation in running activities has considerably increased recently. For example, in the USA, about 19 million runners finished running races in 2013; 17.1 million people competed in 2015 in the USA, and the number of road races increased by 2300 between 2014 and 2015 [28]. The participation of male and female individuals in recreational running in the Czech Republic can be analyzed based on the result lists from a series of running races for amateur runners “Běhej lesy” [29] that take place all over the country. The average ratio of running women in the entire group was about 69%. As age increases, the ratio of female participation tends to decrease. The female ratio was only 35.8% in the oldest category of 56 + . The results of a study by Lee et al. [28] showed that regular running has a positive effect on the cardiovascular, metabolic, musculoskeletal, and neuropsychiatric system, reduces the body fat to body mass ratio, and increases the fat-free mass ratio, as well as muscle mass. Running thus secondarily reduces premature mortality rate by 25–40% and extends life expectancy by up to 3 years compared to nonrunners [30].

The escalating proportion of inactive individuals represents a significant concern within the current adult population. The most recent available European data reveal that the prevalence of physical inactivity in adults in the European Union (EU) exceeds 30% (36.2%, 95% *CI*: 35.1–37.3) [31]. These results come from a cross-sectional survey involving 19,645 people aged 18 to 64. In the Czech Republic, the prevalence of physical inactivity stands at 35.9% (95% *CI*: 32.4–39.5) [31]. One of the contributing factors to physical inactivity is the notable rise in overweight and obesity within the population [3]. According to the World Health Organization (WHO), 39% of adult men and 40% of adult women are overweight, while 11% of men and 15% of women are classified as obese among those aged 18 and older [32].

As running affects the ratio of muscle and fat mass in real time, the question arises whether regular leisure running will have a positive effect on body composition (BC) of runners at an older age and whether the changes in BC caused by aging will be different in runners than in non-active individuals [33, 34].

The primary objective of this study was to investigate the impact of a long-term, regular PA in the form of leisure-time running with the minimal volume of 10 km/week on changes in body mass, body fat, and visceral fat in recreational adult runners both sexes compared to inactive adult individuals of the same age range 18–65 years. The second objective of the study was to determine whether the changes induced by the applied PA regime depend on age and gender.

To explain the above, we formulated a working hypothesis that long-term physically active adults who regularly engage in running for a minimum of 10 km/week have better prospects for PA and significantly better body composition parameters than inactive individuals in all age categories (18–25, 26–35, 36–45, 46–55, 56–65). The changes in body composition parameters depend on age and gender.

## Materials and methods

The study was implemented within an interdisciplinary study with several outcomes that examined mutual relations: Healthy Aging in Industrial Environment Study-Program 4 (4HAIE) (CZ.02.1.01/0.0/0.0/16_019/0000798). The main objective of the 4HAIE study was to assess the effect of air pollution on health, physical activity, and aging. The study focused on the adult population in two regions of the Czech Republic. The Moravian-Silesian Region is an industrial region with increased air pollution, and the South Bohemian Region is a control region where agriculture prevails, with relatively low levels of air pollution. Participants were selected deliberately. The number of participants was also determined with respect to project funding and the measurement capacity of the laboratory.

### Recruitment strategy 4HAIE

For the 4HAIE study, specifically, the recruitment of study participants was commissioned to a professional social science research and marketing company selected through a publicly advertised tender. The sample was collected using quota sampling based on location, age, gender, and PA status (active runner vs. inactive). A detailed list is presented in the article by Elavsky et al. [35]. The study aimed to enroll a total of 1500 participants aged 18 to 65, with 60% being physically active runners and 40% inactive controls. These participants were evenly distributed between two regions: an industrial area with high pollution levels, serving as the experimental group (Moravian-Silesian Region—excluding the district of Bruntál and specific counties in the district of Frýdek-Místek), and a low-pollution region designated as the control group (South Bohemian Region). The selected sample size was determined to be the largest feasible size that would accommodate the cohort’s stratification by age groups and enable the analysis of outcomes targeted by the interdisciplinary teams involved. No formal unifying power analysis was however conducted. Participants were being recruited from the community through various approaches, including in-person recruitment through agency workers and online (using social media, job portals and other websites) and off-line advertisements (billboards, newspaper ads, flyers). Specialized recruitment activities tailored to runners were also carried out, such as presentations and information booths at running races, through running clubs, and at other relevant community events. The final number of participants measured was 1311 due to restrictions imposed in the Czech Republic as part of combating COVID-19. Recruitment efforts commenced in February 2019, with measurements being completed in August 2021). Inclusion and exclusion criteria are presented in Table 1.

**Table 1:** Inclusion and exclusion criteria

**Common criteria**	
**Inclusion criteria**	Aged between 18 and 65 years; non-smoking; able to perform normal physical activity including running (i.e., no medical restrictions on physical activity mandated by a physician); residing in the region for the past 5 years, without any plans to move in the following year; having a smartphone and access to the Internet (Wi-Fi or data)
**Exclusion criteria**	Acute (in the past 6 weeks) medical issues (pain, injury, surgery) preventing normal physical activity, other acute illnesses; pregnancy; radiological examination in the past 7 days; factors that would exclude a graded exercise test or magnetic resonance examination (such as a pacemaker, radioactive or surgical devices/implants, insulin pump)
**Inclusion criteria for runners**	
Running as a main exercise activity, > 150 min of moderate or > 75 min of strenuous physical activity per week (or an equivalent combination of moderate and strenuous physical activity) [36], ≥ 10 km of running per week for at least 6 weeks prior to the tests, intending to continue running for next 12 months, permanent (≥ 5 years) whole-year residency in the determined areas, not planning to move away from the determined areas during the next 12 months, with Internet access, and using a smartphone (with iOS or Android 5.0 or higher)	
**Inclusion criteria for “inactive” individuals**	
< 150 min of moderate or < 75 min of strenuous exercise per week (i.e., not meeting current public physical activity guidelines [36], capable of running, but not running or running irregularly and/or less than 6 weeks prior to the tests, no contraindications to exercise, permanent (≥ 5 years) whole-year residency in the determined areas, not planning to move away from the determined areas during the next 12 months, with Internet access, and using a smartphone (with iOS or Android 5.0 or higher)	

### Procedures and protocol 4 HAIE

**Fig. 1:**
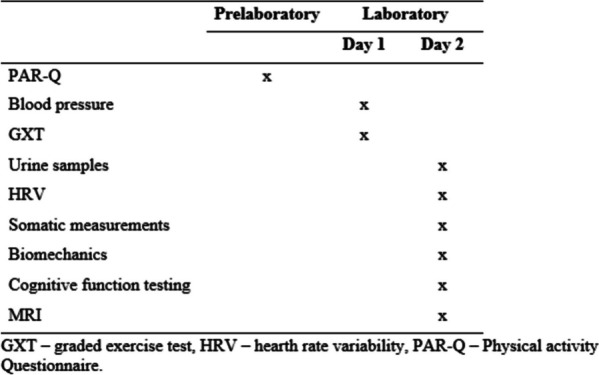
Measurement protocol

Participation in the study was voluntary, and all participants signed a written informed consent agreement that they received during the initial measurements in the laboratory. To safeguard the anonymity of each participant, a unique code label was assigned to them. This code label was used as the reference in all measurement protocols and databases. The laboratory measurement protocol is presented in Fig. 1. The measurement protocol did not include the participants’ dietary analysis. Further information regarding the 4HAIE study’s design, methods, and measurement protocol can be found in previously published works. Physiological and somatic measurements were presented in the publication by Cipryan et al. [37]; the behavioral, psychological, and neuroimaging protocol (MRI structural sequences) was presented in the publication by Elavsky et al. [35]. The study was approved by the Ethics Committee of the University of Ostrava (protocol code OU-87674/90–2018 and date of approval 29 November 2018) and followed the principles of the Helsinki Declaration.

### Study participants

Out of the initial cohort of 1311 individuals (698 males, 613 females) from the 4HAIE study, the current study focuses on 1296 individuals (605 females and 691 males). Fifteen participants (8 females and 7 males) were excluded due to missing data from the DXA body composition measurement. These individuals had implants or metal parts in their bodies, and so their parameter values would be distorted. A detailed description of the participants is presented in Table 2.

**Table 2:** Numbers and basic characteristics of all participants in monitored individuals divided into five groups by age (mean ± SD)

Age categories (years)	Runners				Inactive	
	*n*	Age (years) M ± SD	km/week M ± SD	Min/week M ± SD	*n*	Age (years) M ± SD
Male						
18–25	90	21.2 ± 2.4	22.7 ± 14.6	136.5 ± 118.9	52	21.2 ± 2.3
26–35	111	31.1 ± 2.7	29.6 ± 20.5	170.1 ± 136.1	56	30.1 ± 2.7
36–45	125	40.7 ± 2.5	26.7 ± 18.2	151.3 ± 95.2	57	41.1 ± 2.5
46–55	87	49.5 ± 2.8	31.4 ± 21.4	181.8 ± 115.6	51	50.1 ± 2.9
56–65	24	60.4 ± 2.9	28.3 ± 17.6	189.5 ± 108.2	38	60.6 ± 3.0
Female						
18–25	74	21.2 ± 2.3	22.7 ± 15.0	159.4 ± 137.1	66	21.2 ± 2.3
26–35	55	31.0 ± 2.8	25.9 ± 14.8	152.9 ± 81.7	58	30.0 ± 3.0
36–45	102	41.1 ± 2.9	25.9 ± 14.5	163.3 ± 91.0	59	41.0 ± 2.7
46–55	58	49.5 ± 2.9	21.6 ± 14.0	144.6 ± 110.4	65	49.8 ± 2.8
56–65	13	58.0 ± 2.4	25.4 ± 15.2	175.5 ± 108.7	55	59.5 ± 2.8

*n* Frequency, *M* Mean, *SD* Standard deviation

Table 2 also states the average kilometers and running duration per week for the group of active runners. We obtained the values from the Aerobic Centers Longitudinal Study (ACLS) questionnaire [38]. We analyzed the runners’ episodes per week, number of kilometers, and duration of each episode.

### Somatic measurements

To ensure standard measurement conditions, the participants were admitted to the research center 15 h before measurement (they slept in the sleep laboratory), which allowed us to monitor their behavior and hydration prior to the measurement. Measurements started at 8:30 a.m. the next morning. Participants were measured barefoot, in their underwear. All measurements were taken in the following order: body height (BH), body mass (BM), and body composition. BH and BM, which are the input parameters for the bone densitometer software, were measured using the InBody BSM 370 stadiometer (Biospace, South Korea), which is regularly calibrated. Table 3 presents the BH values without any significant differences between runners and inactive individuals (*p* ˃ 0.05). Additionally, the BH and BM values were used to calculate the body mass index (BMI). The weight status was assessed according to WHO: P1: ≤ 18.49 (kg/m^2^)—underweight, P2: 18.50–24.99 (kg/m^2^)—normal weight, P3: 25.00–29.99 (kg/m^2^)—overweight (pre-obesity), P4: ≥ 30.00 (kg/m^2^)—obesity class. Body composition included body fat (BF) and visceral fat (VF) expressed by area (cm^2^). To measure body composition, the DXA method was used, with a Hologic QDR Horizon A bone densitometer (Hologic, Waltham, MA, USA), which is regularly calibrated and undergoes technical safety control (TSC). The reliability and typical error of measurement were also checked in the densitometer [39]. The positions during measurement and the measured segments were presented in the protocol article [37].

We evaluated the aging stages of reproduction in women. Women classified themselves into the following categories according to a self-assessment: 1—premenopausal, 2—early perimenopausal, 3—late perimenopausal, 4—early postmenopausal, and 5—late postmenopausal [40]. No significant differences were found between the runners and inactive individuals.

### Physical activity assessment

To analyze physical activity, we used the standardized, internationally recognized Aerobic Centers Longitudinal Study (ACLS) questionnaire [38]. The ACLS questionnaire was administered online via the Qualtrics platform. The participants completed a questionnaire prior to the commencement of laboratory measurements; the average time to complete the questionnaire was 30 min.

We analyzed the number of running episodes per week and the duration of each episode in the runners. Based on the values, we calculated the duration of running per week. To quantify running, we also used the records of kilometers run and the duration of each running episode in the week. The values of body composition of runners in terms of the implemented PAs were used as an indirect controllable indicator of questionnaire validity.

### Statistics analysis

The normality of the distribution was verified by the Shapiro–Wilk test. The data did not have a normal distribution; therefore, we used the nonparametric Mann–Whitney *U*-test to assess the differences between the values of runners and inactive individuals and to assess the effect of age. The level of statistical significance for all the used tests was set at *α* = 0.05. Practical significance was assessed using the effect of size *d*. The *d*-value at the level of 0.3 indicates a small effect, 0.3–0.5 a medium effect, and ˃ 0.5 large effect [41]. The value of *d* ˃ 0.3 is considered practically significant. To compare the representation in the individual BMI categories (male, female) and in the categories of the reproduction age stage (female) between runners and inactive individuals, we used the chi-square test for the contingency tables. The internal consequences of all data assessed by the chi-square test were tested using the Bonferroni post hoc test [42]. The statistical processing of the results was performed using IBM SPSS Statistics (Version 24; IBM, Armonk, NY, USA).

## Results

**Fig. 2:**
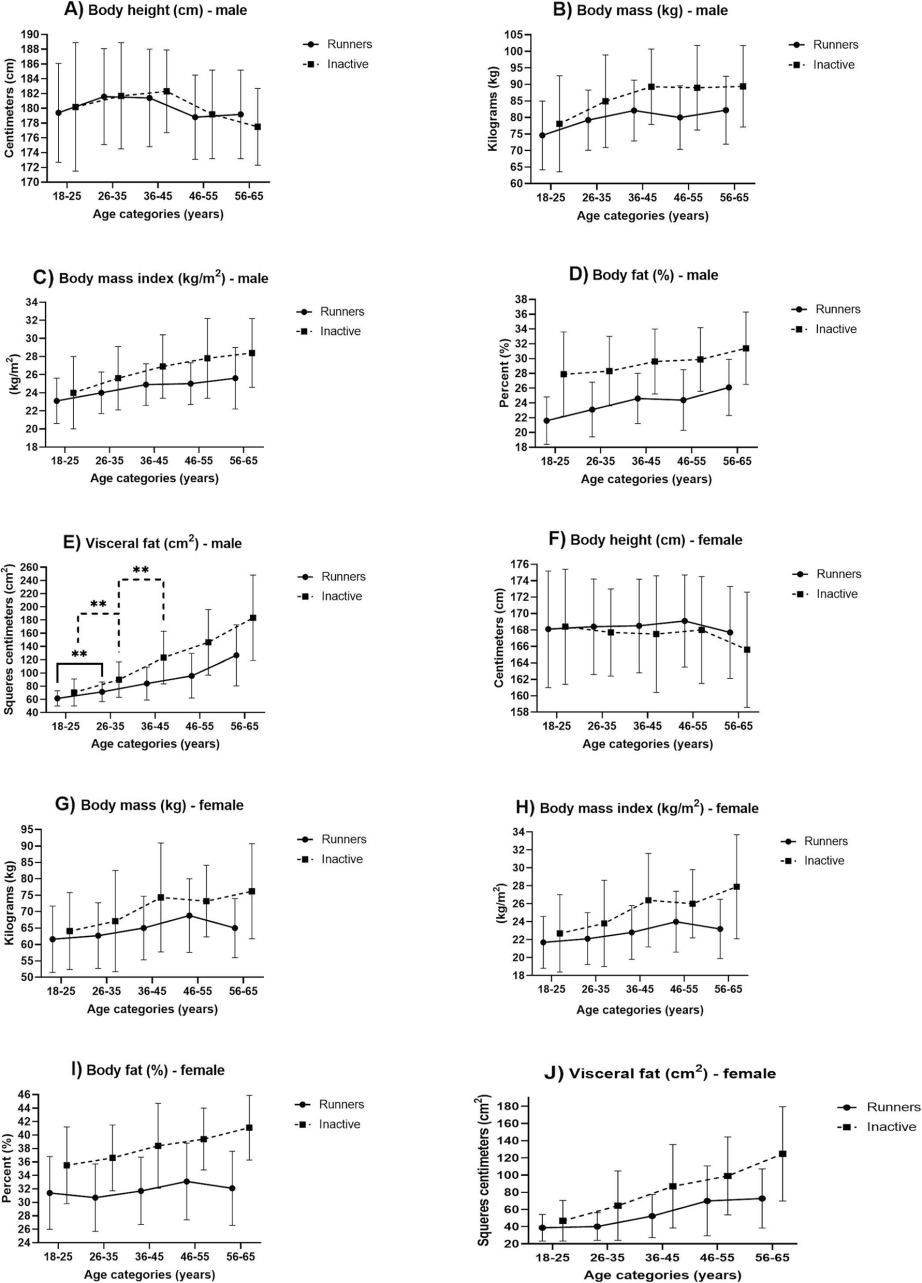
Development of somatic parameters in runners and inactive individuals of different ages

**Fig. 3:**
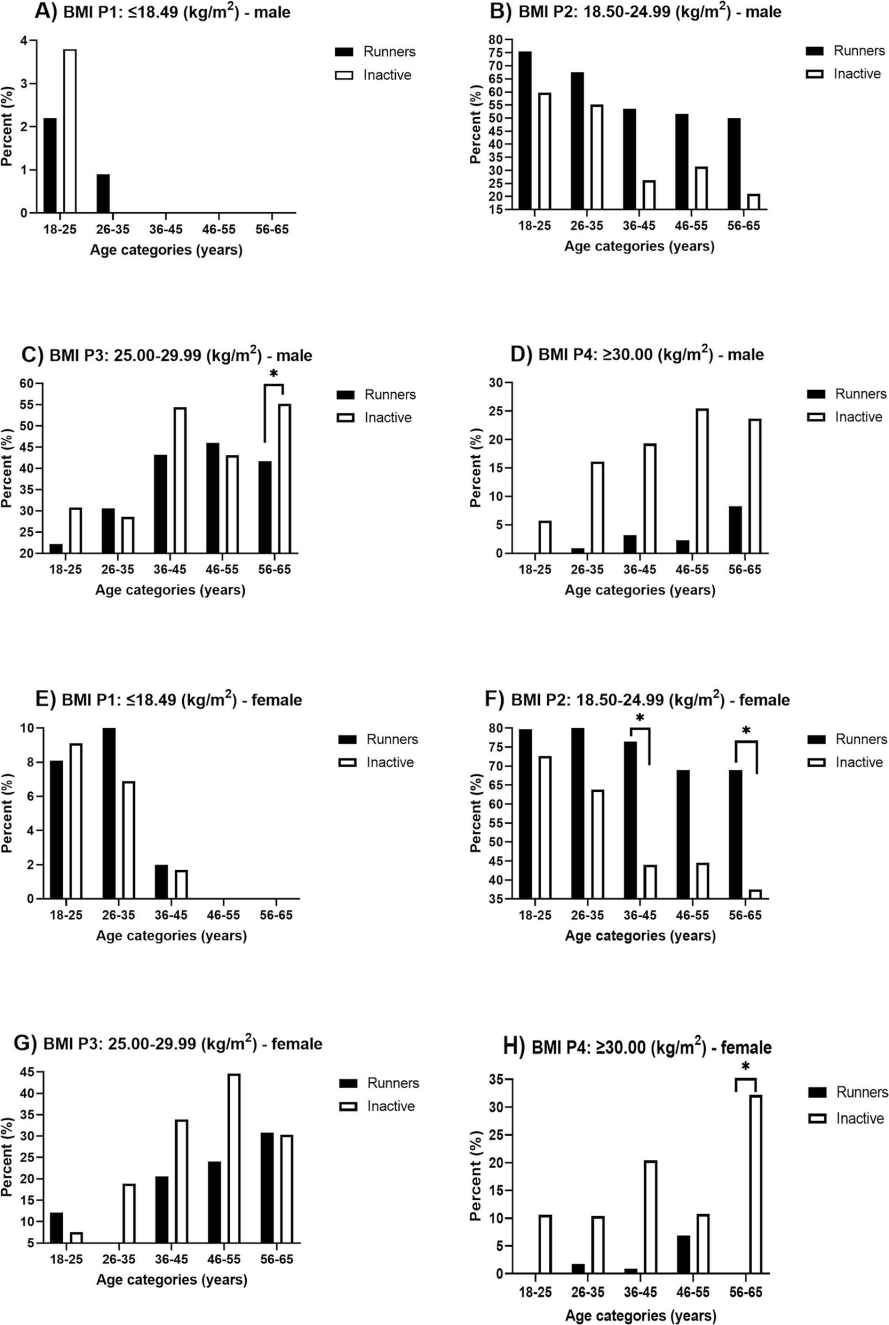
Weight status assessment of the individual monitored groups

The somatic parameters monitored by men and women in the groups of runners and inactive individuals are presented in Fig. 2. Analyses of monitored somatic parameters and their differences in individuals of different ages between runners and inactive individuals, as well as within individual groups, are presented in Fig. 2 and Table 3. Figure 3 shows the assessment of the weight status of the individual monitored groups. When assessing the body (BF) ratio, we only used the relative value expression (percentage) due to the different body mass (BM) of runners and inactive individuals.

Figure 2 presents the development of somatic parameters in runners and inactive individuals of different ages, with significant changes highlighted in each group. Only changes that were statistically significant (*p* < 0.05) and practically significant (*d* > 0.3) are labelled significant. Natural development prevails until 35 years of age; implemented physical training prevails at a later age.

Our analysis revealed a positive relationship between age and somatic parameters (body mass, BMI, body fat, and visceral fat) in both male runners and inactive individuals. With increasing age, these parameters increased gradually. However, only visceral fat showed a significant increase. There was an increase in runners between the age groups of 18–25 and 26–35 (*p* < 0.001, *d* = 0.37). There was a significant increase in inactive individuals between the age groups of 18–25 and 26–35 (*p* < 0.001, *d* = 0.41), as well as between 26–35 and 36–45 (*p* < 0.001, *d* = 0.44).

Our study also found a positive relationship between age and somatic parameters in females. With increasing age, body mass gradually increased, with the exception of the age group 56–65 in runners and 46–55 in inactive individuals. The changes between age categories were consistent for both body mass and body mass index (BMI). The percentage of body fat (BF) also increased continuously with increasing age, with the exception of a decrease observed in runners aged 56 to 65 years. In addition, we observed a continuous increase in visceral fat (VF) in both female groups. However, the changes in BF and VF across age categories were continuous but not statistically significant.

Table 3 presents the differences between the values of the monitored somatic parameters of the runners and inactive individuals in the individual age groups. In the monitored parameters, age dependencies differ between men and women.

**Table 3:** Statistical analysis results—somatic parameters of male and female runners vs inactive participants

Parameters	18–25 years Diff (*d*)	26–35 years Diff (*d*)	36–45 years Diff (*d*)	46–55 years Diff (*d*)	56–65 years Diff (*d*)
Male					
BH (cm)	− 0.8^NS^	− 0.1^NS^	− 0.9NS	− 0.4	+ 1.7^NS^
BM (kg)	− 3.5^NS^	− 5.7* (0.17)	− 7.2*** (0.31)	− 9.0*** (0.38)	− 7.2* (0.30)
BMI (kg/m^2^)	− 0.9^NS^	− 1.6* (0.20)	− 2.0*** (0.31)	− 2.8*** (0.37)	− 2.8** (0.33)
BF (%)	− 6.3*** (0.55)	− 5.2*** (0.50)	− 5.0*** (0.50)	− 5.5*** (0.55)	− 5.3*** (0.51)
VF (cm^2^)	− 9.0* (0.22)	− 18.4*** (0.32)	− 39.2*** (0.48)	− 50.5*** (0.50)	− 56.7** (0.44)
Female					
BH (cm)	− 0.3^NS^	+ 0.7^NS^	+ 1.0^NS^	+ 1.1^NS^	+ 2.1^NS^
BM (kg)	− 2.5^NS^	− 4.4^NS^	− 9.3*** (0.29)	− 4.4* (0.21)	− 11.2** (0.34)
BMI (kg/m^2^)	− 1.0^NS^	− 1.7^NS^	− 3.6*** (0.36)	− 2.0** (0.28)	− 4.7** (0.38)
BF (%)	− 4.1*** (0.33)	− 5.9*** (0.53)	− 6.7*** (0.50)	− 6.3*** (0.53)	− 9.0*** (0.54)
VF (cm^2^)	− 8.1* (0.18)	− 24.2*** (0.38)	− 34.7*** (0.38)	− 29.0*** (0.38)	− 51.9** (0.40)

*BH* Body height, *BM* Body mass, *BMI* Body mass index, *BF* Body fat, *VF* Visceral fat, *Diff* Difference, (*d*) effect size, *NS* Non-significant

^*^p < 0.05, **p < 0.01, ***p < 0.001

When comparing male runners and inactive participants, the results showed that runners in all age categories had a considerably lower body mass (BM), BMI and percentage body fat (BF) ratio. The applied running load has a considerable effect on the visceral fat (VF) value. Differences were statistically and practically significant (*p* < 0.05, *d* > 0.3), except for the values of BM and BMI in the age category of 18–25 (*p* ˃ 0.05). Practical significance was not demonstrated in VF in the age category of 18–25 and in the BM and BMI in the age category of 26–35 years (*d* < 0.3).

When comparing female runners and inactive participants, as well as male participants, no significant differences in body height were observed between runners and inactive participants (*p* > 0.05). Female runners had lower values of body mass (BM), body mass index (BMI), body fat (BF), and visceral fat (VF) than inactive participants in all age categories. However, statistical and practical significance for BM was only observed in the age category of 56–65 years (*p* < 0.05, *d* > 0.3). In BMI, statistical and practical significance was confirmed in the age categories 36–45 and 56–65 (*p* < 0.05, *d* > 0.3). The percentage of BF ratio showed statistical and practical significance in all age categories (*p* < 0.05, *d* > 0.3). Statistical significance was also confirmed in all age categories for VF (*p* < 0.05), but practical significance was not confirmed in the age category of 18–25 years (*d* < 0.3). Physical training is essential in higher age categories as it can considerably slow down physiological aging.

Figure 3 presents the percentage representation of participants in the individual BMI categories and the results of a comparison between the runners and inactive participants.

The underweight category (P1) in the monitored group included both runners (male in the age categories of 18–25 and 26–35 years, female 18–25 to 36–45 years) and inactive individuals (male in the age categories of 18–25 years and female 18–25 to 36–45 years). In all age categories, both female and male runners had a higher percentage representation in the normal weight category (P2) compared to inactive individuals, with a significant difference found in female runners in the age categories of 36–45 and 56–65. In the overweight category (P3), inactive male individuals have a higher percentage share than runners in the age categories of 18–25, 36–45, and 56–65 years. The difference in the age category of 56–65 is significant. Inactive female individuals have a higher percentage share in the P3 category than runners in the age categories of 26–35 to 46–55.

In the obesity category (P4), inactive male and female individuals had a higher share in all age categories than runners. The difference was significant in the female age category of 56–65.

## Discussion

### Comparison of somatic parameters of runners and non-runners

The selected runners were required to complete at least 10 km or running per week. The actual average volume was from 21.6 to 31.4 km/week, and it showed a significant effect on body mass, body fat, and visceral fat. The running volume was implemented by the runners in the long term, and it was a part of their daily routine and thus was not altered as a result of participating in the study. When compared with inactive participants, the runners showed significantly lower values of BF and VF in all age categories. In terms of body mass, running is an effective activity to maintain optimal weight. In all age categories, active runners (both male and female) showed a better weight status compared to inactive individuals. Moreover, a more detailed assessment of weight status revealed a higher proportion of runners of normal weight (P2) and a lower proportion of obese runners (P4) in all age categories compared to inactive individuals (Fig. 3). Diet and dietary habits were not monitored in the study. We operated under the assumption that active individuals adapt their dietary habits to support their PA. Dietary habits remain unchanged [43].

When assessing male runners in all age categories combined, 36.2% were classified as overweight (P3) and 2.1% as obese (P4), indicating considerable differences from population data reported by the World Health Organization (WHO) in 2021, which states that 39% of men are overweight and 11% are obese [32, 44]. Overweight in runners is most likely caused by the higher volume of their muscle mass due to the implemented running, which is documented by the lower values of their BF.

In inactive male individuals in all age categories, there were 41.7% overweight (P3) individuals and 21.7% obese (P4) individuals. The values are higher than the population data [32]. When assessing female runners in all age categories, 16.6% individuals were overweight (P3), and 2.0% were obese (P4). These values are also considerably lower than the population data. The WHO states 40% of overweight women and 15% of obese women. In inactive female individuals in all age categories, 27.0% were overweight (P3) individuals, and 16.5% were obese (P4). The overweight values compared to the population data [32] were considerably lower, and the obesity numbers were higher. Compared to Eurostat, which states 21.4% obese men and 17.7% women in the Czech Republic, the obesity values (P4) in runners (male and female) are considerably lower, and the values of the inactive individuals (male and female) are within the intentions of the values presented [44].

The assessment of PA according to BMI indicates that inactive individuals are more likely to be overweight or obese. However, many authors do not consider using the BMI for obesity assessment to be suitable as it does not reflect the values of visceral fat, body fat, and its distribution [10, 45, 46]. Therefore, it is suitable to use percentile graphs when assessing the adequacy of body mass (especially for children) or to use the more precise body composition evaluation. Other anthropometric measurements can be used for more precise diagnostics (waist circumference or waist-to-hip ratio) to assess of the body fat distribution [47]. However, it is better to use modern methods (BIA, DXA, CT, MRI) that allow quantification of the volume and mass of body tissues, including visceral fat [48–50].

The assessment of the risk of obesity evaluated by BMI was confirmed in the assessment of body fat (BF) and visceral fat (VF). Compared to inactive individuals, runners had significantly lower BF and VF values in all age categories, except for unconfirmed practical significance in VF values in the female age categories of 18–25 years. The oldest age category of male runners (56–65) had mean VF values within the mean values of the inactive male age category of 36–45. The oldest age category of female runners (56–65) had mean VF values corresponding with the inactive female age category of 26–35. In particular, the significantly lower VF values in the monitored runners are significant as VF is metabolically more active and secretes cytokines and hormones that exert metabolic disturbances such as insulin resistance and chronic low-grade inflammation at a higher rate [18]. Therefore, VF is also considered to be a more important risk factor in cardiovascular diseases and obesity than increased BM, BMI, or BF [15–17].

Our results correspond to the conclusions of case studies that deal with the effect of regular aerobic physical activity (PA) on the reduction of adipose tissue and visceral fat. A regular and long-term PA stimulates the blood supply in the fat tissue and mobilizes fat that results in the supply of fatty acids into the skeletal muscles at a rate corresponding to the metabolic requirements [51, 52]. Regular PA increases fat mobilization. Therefore, physically active individuals have relatively low-fat mass and lower VF values that can “slow down” aging. The duration and intensity of the load are fundamental for the actual level of BF and VF [51, 52].

The results of the weight status and body composition assessment of the monitored individuals indicate that a regular PA in the form of recreational running has an effect on the reduction of the prevalence of overweight and obesity and the related secondary medical risks. Obesity is a risk factor in many serious diseases, such as musculoskeletal disability affecting bones; joints and soft tissue [53, 54]; metabolic diseases: diabetes mellitus type 2, metabolic syndrome, and nonalcoholic fatty liver disease [55–61]; and cardiovascular diseases: ischemic heart disease, stroke, heart failure, and hypertension [62–66].

### Changes in somatic parameters in individuals of different ages

All monitored somatic parameters increase with the increasing age of the participants in male runners and inactive individuals. The increase in BM, BMI, and BF is sharper by the age category of 36–45 let (Fig. 2B–D). However, no step differences were found between adjacent age categories. The changes found could thus be labelled as continuous. The slight decrease in the values of BM, BMI, and BF in runners in the age category of 46–55 was probably caused by the higher volume of running load. This age category has the highest running volume (Table 2). A detailed analysis of the differences between the age categories showed the largest difference in the mean values in runners between the age categories of 18–25 and 56–65 (*BM* = 7.6 kg, BMI = 2.5 kg/m^2^, *BF* = 4.5%). The largest difference in mean values in inactive individuals was in mean values between age categories of 18–25 and 56–65 years (*BM* = 11.3 kg, BMI = 4.4 kg/m^2^, BF% = 3.5%). Visceral fat (VF) increased continuously without any significant changes between the age categories starting at 26–35 in runners and 36–45 allowed in inactive individuals (Fig. 2E). The analysis of VF development curve (Fig. 2E) shows a step change that signals an elevated increase in the VF in the age category of 56–65 (runners and inactive). The largest difference in the mean VF values of runners and inactive was in the age categories of 18–25 and 55–65 (runners: 64.6 cm^2^, inactive: 112.7 cm^2^). The described differences in the monitored parameters (BM, BMI, BF, and VF) were not significant, except for changes in VF between the runners’ age categories of 18–25 vs 26–35, and between the inactive individuals’ age categories of 18–25 vs 26–35 years and 26–35 vs 36–45 years. The results show an obvious confrontation between a natural development regression and training in the form of regular leisure-time running with increasing age.

Like in the male category, the monitored somatic parameters increase in both female runners and inactive individuals with increasing age of the participants. However, the BM, BMI, and BF values of runners slightly decrease in the age category of 56–65 (Fig. 2G–I). This could be caused by the higher volume of run kilometers per week, which is the highest of all age groups (Table 2). No significance was confirmed in differences between adjacent age categories; therefore, changes are continuous. The greatest changes in the mean values of BM, BMI, and BF in the runners were found between the age categories of 18–25 and 46–55 (*BM*: 7.2 kg, BMI: 2.3 kg/m^2^, and *BF*: 1.7%). The highest differences in the monitored parameters in the inactive were found in the following groups: 18–25 and 55–65 (*BM*: 12.1 kg, BMI: 5.2 kg/m^2^, and *BF*: 5.6%). Visceral fat (VF) also increased continuously in both runners and inactive individuals from the age category of 18–25 until the age category of 56–65 years. The difference found between age categories also was not significant. The highest difference in VF was found between 18–25 and 56–65. The difference was 34 cm^2^ in runners and 77.8 cm^2^ in inactive individuals. Our results did not confirm the results of the SWAN study [13], according to which there should be a considerable increase in BF in females approximately 2 years prior to the final menstrual period. In terms of average age and menstrual status, our age category of 46–55 falls into this group. When we compared the BF values of the age category (36–45 years), the increase in BF was not significant in runners or in inactive women (Fig. 2I). This difference may be caused by a different type of data. The SWAN study used longitudinal data, and our study used cross-sectional data. Also, individual differences in women’s reproductive profiles, which change from puberty to menopause, must be considered.

Based on the obtained results, the hypothesis was accepted. The measured sample size is sufficiently large, allowing us to generalize the results. An exception is the number of subjects in the oldest age group, 56–65. It may be caused by a lower overall number of people running regularly at that age. This assumption is confirmed by the numbers of runners in this age group taking part in the running competition “Běhej lesy” [29]. The percentage ratio of men aged 56–65 in the total number of men was 6.9% in 2023; there were 2.4% women.

The acquired data align with the WHO’s recommendations for weekly physical activity. This PA took the form of running, with a volume ranging from 21.6 to 31.4, depending on the age category. The individuals engaged in running as a long-term part of their lifestyle, and they were accustomed to it. Therefore, they did not need to make any significant changes to their daily routines during their participation in the study.

### Limitations

This study has several limitations. The first limitation is that this was a cross-sectional study and not a longitudinal study. This limitation is based on the setting of the 4HAIE study, with one of the goals being to establish a cohort of active and inactive individuals, allowing for future analysis of changes in the observed parameters as they age.

The second limitation is that this was a deliberate selection, as designed by the 4HAIE project. The individuals had to meet the selection criteria of the 4HAIE study: location, age, gender, and PA status.

The third limitation involves not having assessed the influence of diet or caloric intake. We are aware that diet is an important factor that may influence body composition, namely, its fat component. However, the 4HAIE project protocol did not include a validated diet assessment.

The fourth limitation is the analysis of running in the group of runners through self-reporting that is exposed to subjective evaluation. However, a standardized instrument was used.

Another limitation includes the number of participants in the oldest age category (56–65), namely in the group of runners. We did not manage to involve more participants in that age category in the 4HAIE project.

### Strengths of the study

A strong point of the study is the fact that a comprehensive sample of both physically active and inactive individuals was measured under precisely defined standard conditions. The participants were admitted to the research center 15 h before the measurements began, allowing us to monitor their behavior prior to the measurements.

Despite the mentioned limitations, it was indeed demonstrated that an achievable volume of PA in the form of recreational running led to significant changes in the observed parameters among recreational runners, thereby improving their prospects for health and physical fitness compared to inactive individuals of the same age group. Although a minimum running volume of 10 km/week was required, the actual average values were higher (ranging from 21.6 to 31.4 km/week). This is likely related to the motivation of the runners. When individuals adhere to a specific type of PA, running in our case, they dedicate a significant portion of their leisure time to this activity. The obtained data will also be utilized in a planned follow-up longitudinal study.

## Conclusions

The study results have shown that recreational running, with a minimum volume of 10 km/week (in reality, the average volume ranged from 21.6 to 31.4 km/week), led to significantly improved body composition values. It can induce significant changes in body mass, body fat, and visceral fat. However, there are differences between men and women in the relationship between changes in body composition parameters. These changes can provide suitable conditions for engaging in PA at an older age, which is crucial for maintaining independence in old age.

An important conclusion is that the observed significant differences could be attributed to the volume of running, which is feasible without altering one’s daily work routine. Running also offers the advantage of being easily accessible compared to other forms of PA, and its implementation does not require special equipment, partners, or the acquisition of specific skills. Recommended levels of PA are significantly exceeded by recreational runners who have chosen to engage in regular PA, often participating in amateur running races for which they prepare.

The results also showed that the body composition evaluation can be used for the evaluation of the effectiveness of the physical routine with a sufficient level of accuracy.

### Supplementary Information

**Additional file 1. **

## Data Availability

All data generated or analyzed during this study are included in this published article.
